# Regular exercise participation improves genomic stability in diabetic patients: an exploratory study to analyse telomere length and DNA damage

**DOI:** 10.1038/s41598-017-04448-4

**Published:** 2017-06-23

**Authors:** Ivan Dimauro, Antonella Sgura, Monica Pittaluga, Fiorenza Magi, Cristina Fantini, Rosa Mancinelli, Antonio Sgadari, Stefania Fulle, Daniela Caporossi

**Affiliations:** 1 Department of Movement, Human and Health Sciences, University of Rome “Foro Italico”, Rome, Italy; 2 Department of Science, University Roma Tre, Rome, Italy; 3 Department of Neuroscience, Imaging and Clinical Sciences, Interuniversity Institute of Miology (IIM), University “G d’Annunzio”, Chieti, Italy; 4 Department of Geriatrics, Gerontology and Physiatry, University Hospital Agostino Gemelli, Catholic University of the Sacred Heart, Rome, Italy

## Abstract

Physical activity has been demonstrated to be effective in the prevention and treatment of different chronic conditions, including type 2 diabetes (T2D). In particular, several studies highlighted how the beneficial effects of physical activity may be related to the stability of the DNA molecule, such as longer telomeric ends. Here we analyze the effect of exercise training on telomere length, spontaneous and H_2_O_2_-induced DNA damage, as well as the apoptosis level in leukocytes from untrained or trained T2D patients *vs*. age-matched control subjects (CS) (57–66 years). Moreover, expression analysis of selected genes belonging to DNA repair systems, cell cycle control, antioxidant and defence systems was performed. Subjects that participated in a regular exercise program showed a longer telomere sequence than untrained counterparts. Moreover, *ex vivo* treatment of leukocytes with H_2_O_2_ highlighted that: (1) oxidative DNA damage induced similar telomere attrition in all groups; (2) in T2D subjects, physical activity seemed to prevent a significant increase of genomic oxidative DNA damage induced by chronic exposure to pro-oxidant stimulus, and (3) decreased the sensitivity of leukocytes to apoptosis. Finally, the gene expression analysis in T2D subjects suggested an adaptive response to prolonged exercise training that improved the response of specific genes.

## Introduction

The biological processes linking aging and disease risk are poorly understood. Still, aging is considered to date as one of the main factors responsible for several complex diseases including cancer, cardiovascular diseases, and diabetes.

Particularly, type 2 diabetes (T2D) has become very prevalent all over the world, with a projected increasing growth rate for the years ahead^1^. The pathophysiological mechanism that underlines diabetic complications suggests oxidative stress as a main factor. The increased oxidative stress in subjects with T2D is a consequence of several abnormalities (hyperglycemia, insulin resistance, hyperinsulinemia, and dyslipidemia) and induces enhanced susceptibility to damage of proteins, lipids and DNA^2^.

Several studies have already demonstrated that the overproduction of reactive oxygen species (ROS) can produce elevated levels of oxidative DNA damage^3–5^, including telomere attrition^6, 7^. The telomere is a region of repetitive nucleotide sequences at the end of each eukaryotic chromosome, which protects them from attrition and damage. Although the relationship between leukocyte telomere length (LTL) and diabetes is still questioned^8^, different studies have shown that T2D individuals have shorter leukocyte telomeres than non-T2D individuals^9, 10^ that may be associated with disease progression^11^. Indeed, the decreased antioxidant capacity described in patients with diabetes results in greater exposure to oxidative stress and subsequent damage to macromolecules (DNA, proteins, lipids), primarily into cells of the circulation, specifically leukocytes^12^. Assuming that mechanisms of DNA damage and repair are similar in different tissue types^13, 14^, leukocytes can serve as an excellent bio-marker because of their half-life and their presence in all body districts. Moreover, given the correlation and synchrony of telomere shortening between somatic cells, to date LTL is used as a proxy of TL in tissues that are affected by aging^15–17^.

Engagement in regular physical activity and increased physical fitness are recommended for the prevention and treatment of diabetes and other pathological conditions^5, 18, 19^. We recently demonstrated that four months of moderate physical training, besides being beneficial to glycemic control, was also effective in improving the redox homeostasis in diabetic patients, lowering the oxidant species production and/or increasing the endogenous antioxidant defenses^20^. In the present study, we aimed to analyse the effect of regular engagement in moderate physical training on telomere length, spontaneous and H_2_O_2_-induced DNA damage, and apoptosis in purified blood leukocytes derived from untrained and trained T2D subjects, compared to age-matched untrained and trained controls. In addition, we examined whether exercise training affected the transcriptional level of a set of genes involved in DNA repairs systems, cell cycle control, as well as antioxidants and defence systems, by comparing untrained and trained T2D patients.

## Results

### Effect of training on basal leukocytes’ telomere length and spontaneous DNA damage in control and diabetic subjects

**Figure 1:**
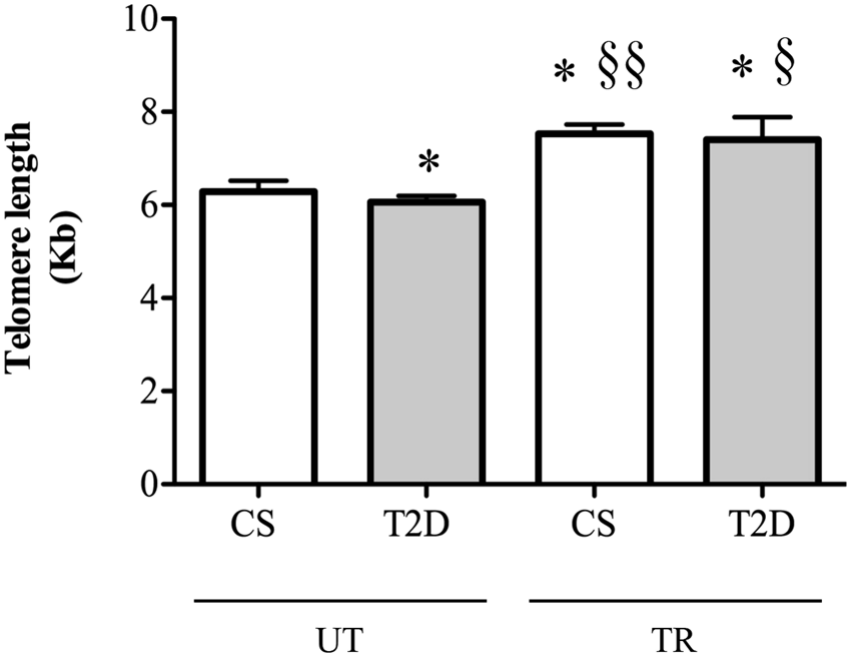
Comparison of leukocytes telomere length in study participants: control subjects (CS) and diabetic patients (T2D) when untrained (UT) and trained (TR) respectively. *p < 0.05 *vs*. UT-CS, ^§§^p < 0.01 *vs*. UT-T2D, ^§^p < 0.05 *vs*. UT-T2D.

Characteristics of subjects participating in the study are shown in Table 1. Figure 1 shows the results from Q-FISH analysis of telomere length in leukocytes of UT-CS and UT-T2D subjects. The statistical analysis revealed a significant difference in LTL between UT groups, the mean telomere length (Kb) in CS was ≈4% longer than in T2D (UT-CS 6.29 ± 0.57 *vs*. UT-T2D 6.06 ± 0.33, P = 0.03) subjects. When analysing the effect of training, all TR subjects exhibited increased telomere lengths than untrained counterparts, with mean averages of both trained groups being significantly greater than in UT groups (≈20–22%). In particular, TR-CS showed a higher average LTL as compared to both UT-CS (TR-CS 7.53 ± 0.49 *vs*. UT-CS 6.29 ± 0.57, P = 0.03) and UT-T2D (TR-CS 7.53 ± 0.49 *vs*. UT-T2D 6.06 ± 0.33, P = 0.01). Similarly, in the TR-T2D group the telomere length was significantly longer compared to both UT-CS (TR-T2D 7.40 ± 1.20 *vs*. UT-CS 6.29 ± 0.57, P = 0.04) and UT-T2D groups (TR-T2D 7.40 ± 1.20 *vs*. UT-T2D 6.06 ± 0.33, P = 0.03). No difference was identified in LTL between the TR groups (TR-CS 7.53 ± 0.49 *vs*. TR-T2D 7.40 ± 1.20, P > 0.05).

**Table 1:** Subject characteristics.

	T2D		CS	
	UT (*n* = *6*)	TR (*n* = *6*)	UT (*n* = *6*)	TR (*n* = *6*)
AGE (yrs)	61.1 ± 3.3	60.1 ± 2.1	62.1 ± 1.3	62.1 ± 4.3
BMI (kg/m^2^)	27.9 ± 2.0	26.9 ± 1.6**	27.8 ± 2.0	27.1 ± 1.3
BG (mg/dL)	116.3 ± 12.5	106.4 ± 6.1*	87.8 ± 8.4	90.3 ± 10.2
HbA1c (%)	6.7 ± 0.7	6.3 ± 0.5*	5.5 ± 0.2	5.4 ± 0.3
Duration of T2D (yrs)	6.2 ± 0.8	6.3 ± 1.2	—	—

**BMI**, Body Mass Index; **BG**, Basal Glycaemia; **HbA1c**, Glycated Haemoglobin; **T2D**, Type 2 diabetes; **CS**, control subjects; **UT**, untrained; **TR**, trained. **p < 0.01 *vs*. UT, *p < 0.05 *vs*. UT. All values are shown as means ± SD.

**Figure 2:**
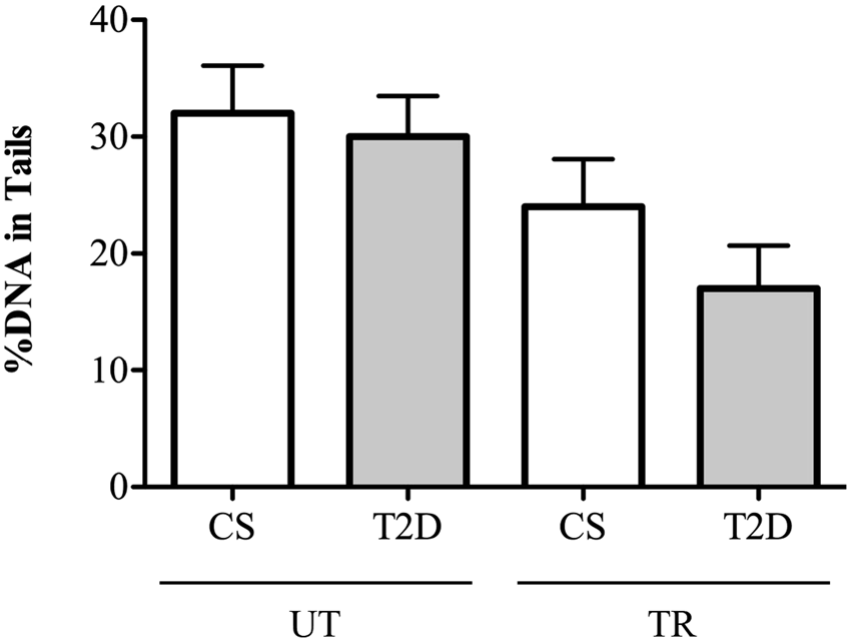
Leukocytes DNA damage in control subjects (CS) and diabetic patients (T2D) of untrained (UT) and trained (TR) groups.

The analysis of spontaneous DNA damage by COMET assay showed no differences in mean average tail values in untrained (UT-CS 32 ± 10 *vs*. UT-T2D 30 ± 8.5, P > 0.05), or trained groups (TR-CS: 24 ± 10 *vs*. TR-T2D: 17 ± 9, P > 0.05) (Fig. 2). Although the difference in DNA damage was not significant, the trained groups showed a trend toward a decrease in spontaneous DNA damage as compared to untrained subjects (P > 0.05).

### Effect of training on H_2_O_2_-induced telomere shortening and DNA damage in leukocytes from control and diabetic subjects

**Figure 3:**
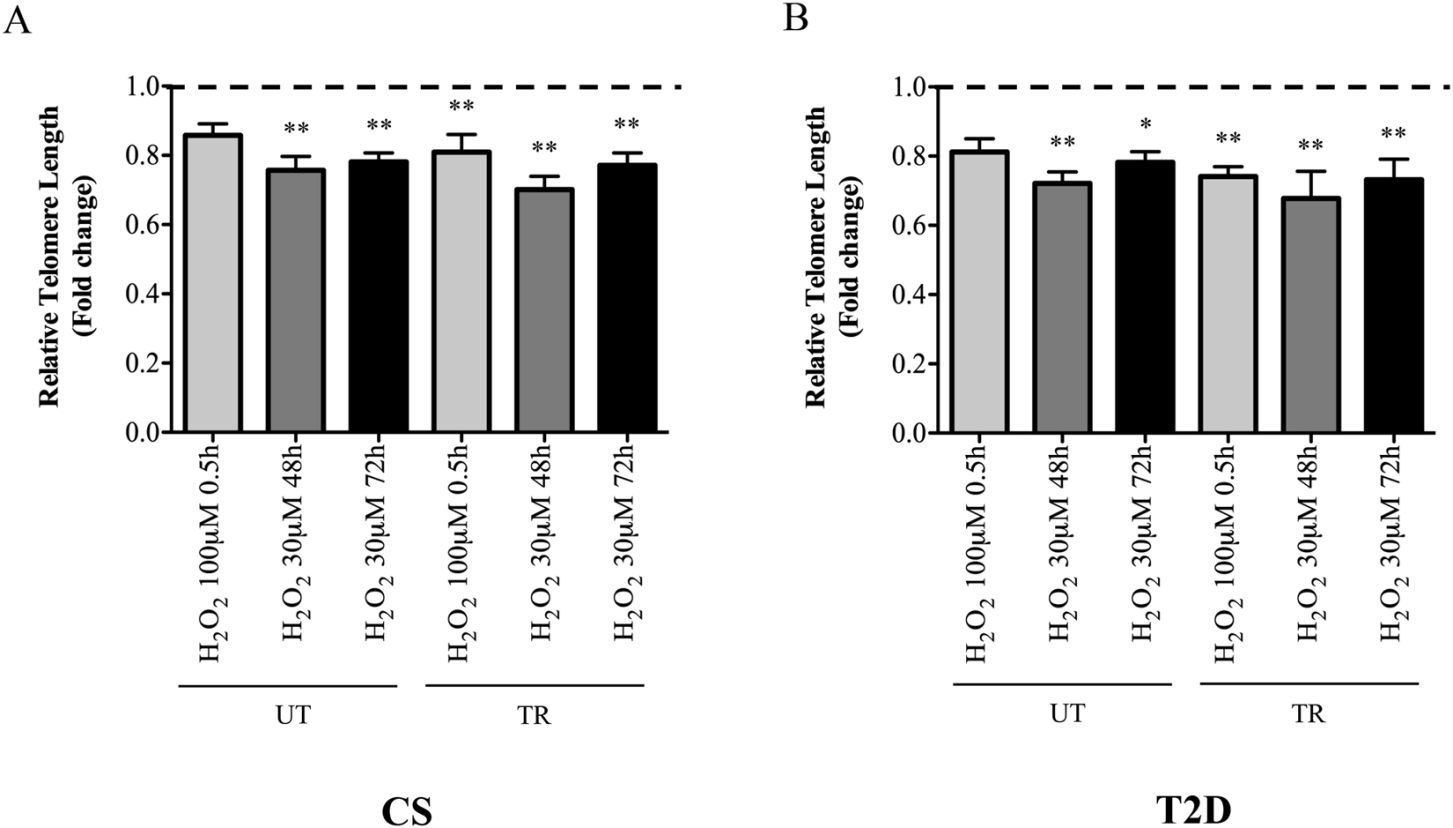
Fold change decrease of telomere length relative to untreated cells in leukocytes of untrained and trained controls (UT-CS: H_2_O_2(0.5h)_ 0.86 ± 0.08; H_2_O_2(48h)_ 0.76 ± 0.1; H_2_O_2(72h)_ 0.78 ± 0.06; TR-CS: H_2_O_2(0.5h)_ 0.81 ± 0.13; H_2_O_2(48h)_ 0.70 ± 0.09; H_2_O_2(72h)_ 0.77 ± 0.09) (**3A**) or untrained and trained diabetic patients (UT-T2D: H_2_O_2(0.5h)_ 0.81 ± 0.09; H_2_O_2(48h)_ 0.72 ± 0.08; H_2_O_2(72h)_ 0.78 ± 0.07; TR-T2D: H_2_O_2(0.5h)_ 0.74 ± 0.07; H_2_O_2(48h)_ 0.68 ± 0.19; H_2_O_2(72h)_, 0.73 ± 0.15) (**3B**). **p < 0.01 and *p < 0.05 vs. corresponding untreated cells. Dashed horizontal line represents no change in telomere length.

The Two-Way ANOVA indicated that an *ex vivo* treatment of leukocytes with H_2_O_2_ induced a similar effect on telomere length in both CS and T2D patients, (Fig. 3A and B). In comparison with untreated cells, all chronic treatments with H_2_O_2_ 30 μM (48 and 72 h) induced a significant LTL (Kb) decrease of between 24% and 35% in both control and diabetic subjects (UT-CS: Untreated, 6.29 ± 0.57 *vs*. H_2_O_2(48h)_, 4.73 ± 0.40, P = 0.0003; *vs*. H_2_O_2(72h)_, 4.91 ± 0.49, P = 0.001; TR-CS: Untreated, 7.53 ± 0.49 *vs*. H_2_O_2(48h)_, 5.26 ± 0.63, P = 0.0001; *vs*. H_2_O_2(72h)_, 5.78 ± 0.53, P = 0.0001; UT-T2D: Untreated, 6.06 ± 0.33 *vs*. H_2_O_2(48h)_, 4.38 ± 0.67, P = 0.0002; *vs*. H_2_O_2(72h)_, 4.73 ± 0.42, P = 0.04; TR-T2D: Untreated, 7.40 ± 1.20 *vs*. H_2_O_2(48h)_, 4.85 ± 0.82, P = 0.001; *vs*. H_2_O_2(72h)_, 5.29 ± 0.51, P = 0.002) (Fig. 3A and B). The acute treatment of H_2_O_2_ 100 μM for 0.5 h induced a slight decrease in LTL, significance was only found in the trained groups, of both control and T2D (TR-CS: Untreated, 7.53 ± 0.49 *vs*. H_2_O_2(0.5h)_: 6.07 ± 0.79, P = 0.003; TR-T2D: Untreated, 7.40 ± 1.20 *vs*. H_2_O_2(0.5h)_: 5.42 ± 0.44, P = 0.003) (Fig. 3A and B). No differences between and within groups were identified in terms of fold decrease in telomere length in response to acute or chronic H_2_O_2_ treatment (P > 0.05).

**Figure 4:**
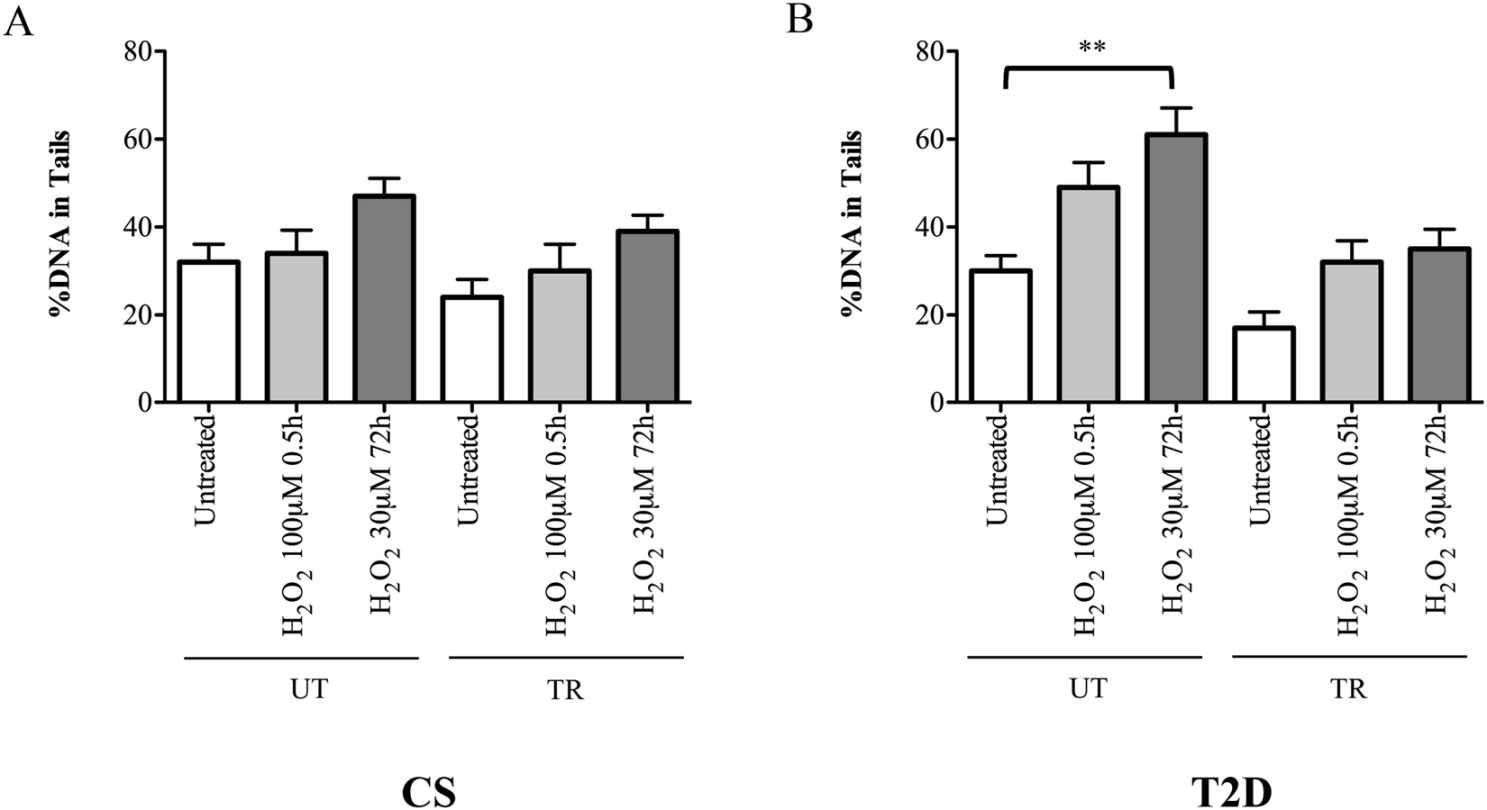
DNA damage analysis in leukocytes of (A) control (CS) untrained (UT) and trained (TR), and (B) diabetic patients (T2D) UT and TR, following an acute (100 μM for 0.5 hrs) or a chronic (30 μM for 72 h) treatment with H_2_O_2_. **p < 0.01.

A different result was found to the induction of DNA damage, where the statistical analysis failed to show any significant difference within and between groups, except for an exercise-disease interaction in untrained T2D subjects (Fig. 4A and B). Indeed, the Two-Way ANOVA analysis demonstrated a significant increase in DNA damage with respect to untreated values in leukocytes from the UT-T2D group exposed to 72 h of 30 μM H_2_O_2_ (UT-T2D: Untreated, 30 ± 8.5 *vs*. H_2_O_2(72h)_, 61 ± 15; P = 0.001). On the contrary, the sensitivity to oxidative DNA damage induced by H_2_O_2_ in leukocytes from TR-T2D was similar to that showed in leukocytes from untrained and trained controls (TR-T2D: Untreated: 17 ± 9; H_2_O_2(0.5h)_, 32 ± 6; H_2_O_2(72h)_, 35 ± 11; UT-CS: Untreated, 32 ± 10; H_2_O_2(0.5h)_, 34 ± 13; H_2_O_2(72h)_, 47 ± 10; P > 0.05; TR-CS: Untreated 24 ± 10, H_2_O_2(0.5h)_: 30 ± 15, H_2_O_2(72h)_: 39 ± 9), always not significant (P > 0.05).

### Effect of training on H_2_O_2_-induced apoptosis in leukocytes from diabetic subjects

**Figure 5:**
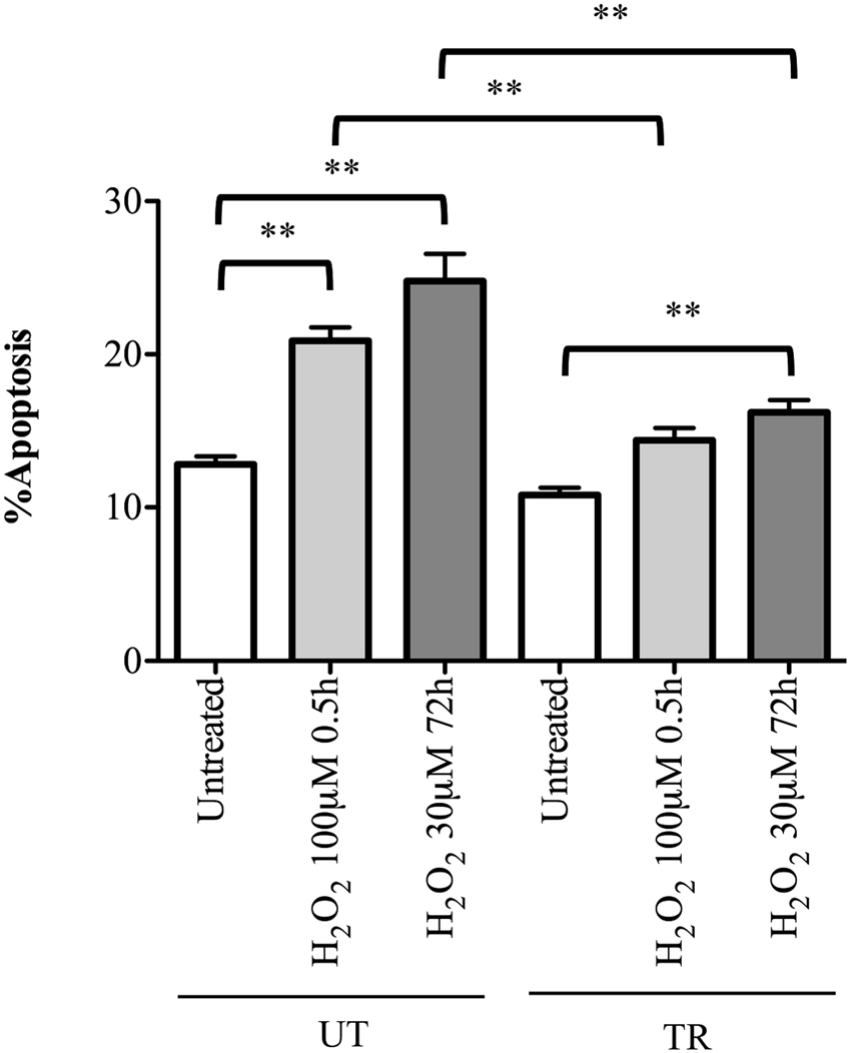
Apoptosis analysis in leukocytes of diabetic patients (T2D) trained (TR) and untrained (UT) following an acute (100 μM for 0.5 hrs) or chronic (30 μM for 72 h) treatment with H_2_O_2_. Apoptosis percentage was evaluated by scoring the number of TUNEL positive nuclei on at least 1000 cells analyzed ×100. **p < 0.01.

To further verify the effect of training and the sensitivity to oxidative stress of diabetic subjects, we analysed the H_2_O_2_-induction of apoptosis in leukocytes from UT- and TR-T2D at the same experimental points utilized for the COMET assay analysis. We found that chronic treatment for 72 h with H_2_O_2_ (30 μM) induced a significant increase of apoptotic cells in both UT- and TR-T2D groups (UT-T2D: Untreated, 12.8 ± 1.3 *vs*. H_2_O_2(72h)_, 24.8 ± 4.3, P = 0.0001; TR-T2D: Untreated, 10 ± 1.2 *vs*. H_2_O_2(72h)_, 16.2 ± 2.0, P = 0.0002), while after acute exposure to 0.5 h with H_2_O_2_ 100 μM, only the UT-T2D subjects showed a significant induction of apoptosis (UT-T2D: Untreated, 12.8 ± 1.3 *vs*. H_2_O_2(0.5h)_, 20.9 ± 2.1, P = 0.0001; TR-T2D: Untreated, 10 ± 1.2 *vs*. H_2_O_2(0.5h)_, 14.4 ± 1.9, P > 0.05). Moreover, the percentage of TUNEL positive cells induced by H_2_O_2_ in UT-T2D subjects was always significantly higher than in TR-T2D subjects, for both acute and chronic treatments (H_2_O_2(0.5h)_, P = 0.0002, H_2_O_2(72h)_, P = 0.001) (Fig. 5).

### Gene expression analysis in leukocytes of trained and untrained diabetic patients

Table 2 summarizes our findings with respect to the detectable genes in an RT-qPCR-array. With respect to significantly regulated genes, out of the 46 genes tested, trained T2D showed a decreased expression of both Uracil-DNA Glycosylase (UNG) (fold change 0.38, P = 0.04), and Oxidation Resistance 1 (OXR1) (fold change 0.35, P = 0.006), as well as an increased expression of H2A Histone Family Member X (H2AFX) (fold change 1.93, P = 0.04) and Growth Arrest And DNA Damage 45 (GADD45) (fold change 3.40, P = 0.003) (Table 2).

**Table 2:** Gene expression analysis in human leukocytes.

Cellular Pathway	Gene	T2D TR *vs*. UT (*Fold changes*)
Base Excision Repair	APEX1	*0*.*84*
	FEN1	*0*.*83*
	LIG1	*0*.*76*
	LIG3	*0*.*67*
	MPG	*0*.*70*
	OGG1	*0*.*53*
	PARP1	*0*.*66*
	PCNA	*1*.*02*
	PNKP	*0*.*67*
	POLB	*1*.*41*
	TDG	*0*.*87*
	UNG	*0*.38*
	CCNO	*N*/*A*
	XRCC1	*0*.*81*
Damaged DNA Binding	BRCA1	*N*/*A*
	ERCC1	*0*.*81*
	ERCC2	*0*.*74*
	H2AFX	*1*.93*
	MSH2	*0*.*56*
	MSH3	*0*.*56*
	RAD1	*0*.*93*
	RAD51	*0*.*65*
	RAD51C	*1*.*19*
	XRCC2	*N/A*
	XPA	*0*.*60*
	XPC	*0*.*70*
	MUTYH	*0*.*98*
	NTHL1	*0*.*53*
Double Strand Breaks Repair	BRCA2	*0*.*75*
	MRE11	*0*.*51*
	PRKDC	*0*.*73*
	RAD52	*0*.*76*
	XRCC6	*0*.*95*
Cell Cycle Checkpoints and Apoptosis	TP53	*0*.*70*
	ATM	*0*.*59*
	BAX	*0*.*89*
	BCL2	*0*.*91*
	CASP3	*0*.*76*
	CHECK1	*1*.*29*
	GADD45	*3*.40**
Antioxidants and Defence Systems	CAT	*0*.*71*
	SOD1	*1*.*05*
	GPX1	*0*.*59*
	GSR	*0*.*72*
	OXR1	*0*.35**
	HSPA4	*0*.*72*

Calculated fold changes of 46 PCR gene products derived from RT-qPCR-array. *p < 0.05, **p < 0.01. **T2D**, Type 2 Diabetes; **UT**, untrained; **TR**, trained; **N/A**, not available.

## Discussion

Based on available data, it is still unclear if a faster rate of telomere attrition and the consequent premature cell senescence can be a cause or a consequence of type 2 diabetes^8^. Although telomere length in different cell types may better reflect specific diseases, tissue-specific aging, or cell-specific adaptations, several studies have shown not only a significant association between LTL shortening and T2D^10^, but also a correlation with time of onset, duration of disease and increasing number of diabetes related complications^6, 21–23^. Indeed, the attrition of this chromosome region seems to be attenuated in patients with well-controlled diabetes^24^. Therefore, telomere shortening in leukocytes may correspond to a similar shortening of telomeres in organs and tissues such as islet β-cells, which lead to premature senescence and subsequent impaired insulin secretion and glucose tolerance^25, 26^.

On the other hand, many studies show that physical activity seems to confer a beneficial effect on LTL maintenance in healthy and diseased elderly people^19, 27–31^.

In 2012, Hovatta and colleagues^32^ reported that subjects with impaired glucose tolerance, who received an intensive lifestyle intervention (i.e., dietary advice, guidance to increase their level of physical activity), showed an increase in the TL over the 4.5 year follow-up period. However, the lack of a population-based control group without any intervention makes the contribution of physical activity difficult to identify. In this exploratory study, we highlight how regular participation in a supervised exercise program determined a significant reduction of telomere attrition in both control and T2D subjects. In addition, while untrained diabetic subjects showed shorter telomeres (−4%) with respect to untrained CS, no differences were observed between trained groups, irrespective of their diabetic or healthy status. Thus, besides confirming the correlation between shorter LTL and diabetes^9, 10^, our results argue in favor of a clear and beneficial role of regular exercise training in counteracting both aging- and T2D-related telomere shortening. Similarly to previous papers analyzing age-matched groups, we found that our training regime preserved about 20% and 22% of LTL in control and T2D subjects, respectively^33, 34^.

Since leukocyte DNA from subjects with T2D is also characterized by increased susceptibility to oxidative damage^6^, and high levels of oxidative stress are known to shorten telomeres^7, 35^, one plausible and potentially testable explanation resided in the capacity of a regular, long-lasting training regime to buffer telomere shortening by affecting the balance between oxidative stress and antioxidants^28, 29^.

According to our previous finding^20^, and differently from the LTL data, no differences were detected in spontaneous DNA damage between UT-CS and UT-T2D groups. It is known that DNA damage measured by the COMET assay in leukocytes correlates with very poor glycaemic control^36^ and our result is not surprising when considering the T2D patients recruited in this study, as well as those who participated in other studies, showed a stabilized glycaemic control^37^. Nevertheless, the training regime produced a decrease, although not significant, in spontaneous DNA damage in both trained groups, possibly due to a decrease of the pro-oxidant cellular environment and increase of endogenous antioxidant defences induced in both diabetic and non-diabetic subjects by this type of exercise, as recently demonstrated by our group^20^.

Physical activity has demonstrated an induction of an adaptive response of several stress-proteins/molecules (anti-oxidants, heat shock proteins)^38–42^, and thereby could modify the susceptibility to subsequent pro-oxidant stimuli^14, 28, 43^. Our results from *ex vivo* treatment with exogenous hydrogen peroxide seem to verify the hypothesis that telomere attrition is dependent upon oxidative damage. Indeed, H_2_O_2_ exposure induces a similar LTL decrease in control and T2D subjects. Although we did not find differences in the telomere sensitivity to H_2_O_2_-shortening between and within groups, we cannot exclude the possibility that longer periods of exposure and/or different concentrations of hydrogen peroxide may determine a different sensitivity that depends on the length of telomere ends.

Differently from LTL, DNA damage evaluated by COMET assay highlighted an enhanced sensitivity to chronic oxidative stress exposure in untrained T2D subjects as compared to those that were trained.

It is known that cellular DNA response to genotoxic stress depends on a combination of different factors, such as the nature of the stress, DNA repair efficiency, presence of telomerase, length of telomeres, and others^44^. In this study, exposure to ROS can induce chemical alterations anywhere in the genome and to the free-nucleotide pools^45^. One form of DNA damage induced by oxidative stress is the alteration of DNA bases, including the common lesion 8-oxoguanine (8-oxoG). It has been recently demonstrated that 8-oxoG has a dual role in inhibiting or stimulating telomerase, depending on whether the free dNTPs are oxidized and inserted during extension, or the telomeric DNA is directly oxidized by free radicals^45^. In particular, the authors proposed that under normoxic conditions unrepaired 8-oxoG lesions promoted telomere lengthening, whereas oxidative stress and pro-oxidant conditions promoted telomere dysfunction and shortening^7, 35, 45^. Considering also that telomeres are particularly prone to oxidative damage at a GGG sequence compared with the rest of chromosomal DNA^35, 46^, and are repaired less efficiently than the rest of the genome^47^, it is thus possible that experimental conditions here resulted in oxidative stress-induced single- and double-strand DNA breaks that were translated preferentially into accelerated leukocytes telomere shortening^48^.

While telomere length resulted significantly decreased in UT-T2D *vs*. UT-CS, no difference was detected in DNA damage. Actually, the COMET assay highlighted that the %DNA in tail was significantly increased only in leukocytes of UT-T2D subjects exposed chronically to H_2_O_2_. An increased susceptibility to oxidative DNA damage in T2D is well recognized^3^; however, it is interesting to note that %DNA in tail was similar between trained subjects, irrespective of their diabetic or non-diabetic status. In agreement with our previous research^20^, we can reasonably assume that the adaptive mechanisms (i.e. increasing endogenous antioxidant defence) induced by regular physical activity may be responsible for a significant reduction of the oxidative DNA damage induced by H_2_O_2_.

The DNA damage detected by COMET assay represents a steady state between induction of lesions and their repair^49^. Therefore, our results cannot exclude the possibility of differences in DNA repair efficiency between trained and untrained T2D subjects. Nevertheless, it is known that genomes cannot be uniformly repaired and that some genomic loci, such as telomeric tracts, resist DNA-damage repair despite a global cellular competence for DNA repair. In particular, experiments conducted by Fumagalli *et al*.^50^ confirm the preferential localization of persistent DNA damage response (DDR) at telomeres and that even long telomeres may be a target for the accumulation of irreparable DNA damage. Therefore, DDR activation either at critically short telomeres or caused by persistent telomeric DNA damage represents the trigger of replicative cellular senescence or apoptosis^48, 50^. The analysis of apoptosis by TUNEL assay showed that leukocytes from untrained T2D subjects were more sensitive to H_2_O_2_-induced cell death than leukocytes from trained T2D subjects, both after acute or chronic exposure. A previous finding showed that moderate exercise attenuates induction of leukocyte apoptosis by oxidative stress by improving the intracellular anti-oxidative capacity^51^. Although we cannot exclude the participation of other stress response proteins, we propose that the capacity of this exercise protocol to improve the status of redox homeostasis could be a main mechanism for the preventative role of physical activity in spontaneous and/or H_2_O_2_-induced apoptosis in leukocytes of T2D patients^52^.

It is also interesting to note that, although in leukocytes of trained T2D patients the treatment with H_2_O_2_ did not induce a significant change of %DNA in tail, a statistical increase of apoptosis was observed in the leukocytes from the same subjects following the chronic exposure to oxidative stress. A possible explanation is that the oxidative DNA damage induced by a chronic exposure to H_2_O_2_, mainly targeted telomere sequences, and overcomes all adaptive mechanisms induced by physical activity to prevent DNA damage. Therefore, chronic exposure to oxidative stress could compromise the capability of leukocyte cells to withstand damage, thereby inducing a persistent telomeric DNA damage response leading to cellular apoptosis^50^. Nevertheless, apoptosis levels in leukocytes of trained subjects were significantly lower than those found in leukocytes of untrained subjects, both following acute or chronic exposure to ROS.

Despite ROS produced by hydrogen peroxide representing one of the main stressors inflicted by endogenous processes *in vivo* ^53^, we have to consider that several other factors could be contributing to increased DNA oxidative susceptibility in the T2D group. In particular, the presence of non-enzymic glycation of DNA bases^54^, differences in paraoxonase activity^55^, generation of lipid peroxidation products^56^, and elevated glucose concentration^57^ have been shown to be associated with DNA damage.

However, the results obtained here by our acute and chronic treatment with hydrogen peroxide, suggest that: (1) irrespective of the original length of telomeric ends, leukocytes exhibit as a main outcome of the oxidative DNA damage a similar telomere attrition in all groups; (2) in T2D subjects, physical activity seemed to prevent a significant increase of genomic oxidative DNA damage (%DNA in tails) induced by a chronic exposure to pro-oxidant stimuli and (3) a decreased sensitivity to apoptosis in H_2_O_2_-treated leukocytes.

To better understand the beneficial effect of moderate, regular exercise in counteracting oxidative stress susceptibility of T2D subjects, we analysed in leukocytes from untrained and trained diabetic patients the expression of genes belonging to signalling pathways related to DNA repair systems, cell cycle control, as well as to antioxidants and defence systems. Out of 46 genes tested, only the expression of 4 genes was significantly modulated in TR-T2D patients *vs*. UT-T2D: specifically, growth arrest and DNA damage (GADD45) and histone H2A (H2AFX) genes were up-regulated, while uracil-DNA glycosylase (UNG) and Oxidation Resistance 1 (OXR1) were down-regulated.

It is know that GADD45 is considered a “stress sensor” to physiological or environmental stressors and it is involved in cell cycle arrest, DNA repair, cell survival and senescence, or apoptosis^58^. Moreover, GADD45 function in either cell survival or apoptosis depends on the extent of cellular/DNA damage in a given cell type that dictates its association with particular partner proteins^59^. Regarding the H2AFX gene, evidence suggests that this protein might serve as a docking site for DNA damage/repair proteins and functions to promote double-strand break repair and genome stability^60^. Similarly to other DNA glycosylases, UNG protein recognizes a damaged or inappropriate base both in a base pair, in a mismatch, and even in single-stranded DNA^61^, whereas OXR1 protein provides protection against oxidative DNA damage produced by endogenous and exogenous oxidative agents^62^.

Although this result requires further investigation, it is in line with the hypothesis that the outcome of the exercise program used in T2D subjects here results in a better management of oxidative stress through the regulation of genes involved in the intricate pathway of the oxidative DNA damage response. In particular, the increased expression of GADD45 and H2AFX could reflect both a greater genomic stability and a more prompt response to oxidative DNA damage of leukocytes from trained subjects with a consequent lower susceptibility to apoptosis^63, 64^, while the expression of genes related to DNA damage recognition and protection from oxidative DNA damage (UNG and OXR1) would be down-regulated^65^.

### Study strength, limitations and conclusions

Given the importance of DNA stability in cellular functions, our findings may have broad relevance highlighting the role of physical activity during physiological and pathological aging. This study represents a new piece of the puzzle proving the beneficial effects of prolonged exercise training on DNA stability in T2D patients. In particular, our results suggest that exercise training may regulate the telomere length in control and T2D subjects and reduce diabetic patients susceptibility to genomic DNA damage induced by a chronic pro-oxidant stimulus.

Although these are preliminary findings and should be interpreted with caution, here we present an exploratory study where a well-trained group of T2D patients were compared to a cohort of age-matched control subjects. Except for exercise training status, the characteristics of subjects within experimental groups were comparable. Moreover, the exercise intervention was well structured (type, frequency, intensity) and supervised from experts to ensure its correct execution from each participant.

At the start of study, we did not have information about the effect size and therefore any sample size estimation and power calculation as an *a priori* have been performed. However, we report difference in mean telomere length with 90% confidence interval among groups because *a priori* power estimations are immaterial at the end of study, and it is the size of effect estimates and the width of the confidence interval that is important^66^. Nonetheless, the small sample size and the presence of only male participants prevent the possibility of making adjustments for confounding variables, representing a limitation of the study.

In conclusion, we strongly believe that the prevention of age-related diseases is an adaptation conferred by engagement in regular exercise training. To date the molecular mechanisms are still poorly understood, thus analysis on specific pathways such as telomerase activity, shelterin complex as well as epigenetic modifications and non-coding RNAs are recommended. For this reason, further studies are needed to establish those molecular mediators by which a regular exercise ensures a “healthy ageing”.

## Material and Methods

### Study design

A total of 24 male subjects, 12 with type 2 diabetes (T2D, 62.1 ± 4.3 years) and 12 as “control subjects” (CS, 61.7 ± 3.9 years) have been recruited for this study at the Fitness Center for the Aged, Department of Gerontological, Geriatric and Physiatric Sciences, Catholic University of Sacred Heart. On the basis of the actual training status, both T2D and CS were divided in untrained (UT) and trained (TR) groups (Table 1). Specifically, 6 T2D and 6 CS have been practicing a supervised long-term, moderate physical training (three times/week of 30 min moderate aerobic activity and 30 min callisthenic gym), for at least one year, while the 6 T2D and 6 CS subjects included in untrained groups were those that have never participated in any training program in the last 5 years.

All participants were fully informed of the research design and associated benefits and risks of the investigation before signing an informed consent approved by the Medical Ethics Committee of the University of Sacred Heart. All experimental protocols were approved by the University of Sacred Heart, and performed in accordance with their guidelines.

Inclusion criteria for the diabetic patients selection were at least 5 years disease duration and therapy with metformin alone or in combination with repaglinide or gliclazide. Exclusion criteria were presence of metabolic unbalance (HbA1c ≥ 7.5), high level neuropathy, high level vasculopathy, cutaneous ulcers, and insulin therapy.

For all subjects, exclusion criteria were as follows: obesity (BMI ≥ 30), severe cardiovascular diseases (ischemic heart disease or heart failure), chronic obstructive pulmonary disease, severe hypertension (diastolic blood pressure ≥100, systolic blood pressure ≥200), smoke habits, alcohol abuse, RX treatments, or diagnostics in the 3 months previous to the blood sampling.

### Leukocytes isolation and treatment

Fasted blood samples were collected in presence of anticoagulants (EDTA or heparin) from all trained and untrained T2D and CS subjects. Peripheral blood leukocytes were isolated by density-gradient centrifugation of whole blood on Ficoll-Paque (Amersham Biosciences). After centrifugation at 1800 rpm for 30 min at room temperature, leukocytes were collected from the interface, rinsed twice in phosphate buffered saline (PBS 1×) (EuroClone) and re-suspended in RPMI 1640 medium with L-Glutamine (EuroClone) with antibiotics Penicillin (100 U/mL)/Streptomycin (0.1 mg/mL) (Sigma-Aldrich) (no serum). Leukocytes were cultured at density of 5 × 10^5^ cells/mL, in RMPI medium supplemented with 10% fetal calf serum FBS (Gibco) and 2% phytohemagglutinin PHA (Gibco). Cell cultures were grown for 48 or 72 h at 37 °C under a 5% CO_2_ atmosphere and 85% air. A dilution of 10 mM hydrogen peroxide (H_2_O_2_) (Sigma-Aldrich, St. Louis, MO) was prepared in PBS immediately before use and then added to medium culture at final concentration of 100 μM (acute treatment) or 30 μM (chronic treatment). For chronic treatment, medium culture with H_2_O_2_ 30 μM was replaced every 24 h for 2 days (48 h treatment) or 3 days (72 h treatment); for acute treatment, cells grown for 24 h were collected by centrifugation, exposed to H_2_O_2_ 100 μM for 0.5 h in fresh medium), rinsed in PBS and re-suspended in conditioned, drug-free, medium for additional 24 h. From each culture, three different aliquots have been collected and processed independently for telomere, COMET and apoptosis analyses. All cultures have been performed in duplicate and proper untreated cultures have been settled for each experimental point.

### Measurement of telomere length: Quantitative-Fluorescent *In Situ* Hybridization (Q-FISH)

To evaluate telomere length in untreated as well as in H_2_O_2_ treated leukocytes (100 μM for 0.5 h or 30 μM for 48 h and 72 h), Quantitative Fluorescence *in Situ* Hybridization (Q-FISH) analysis was performed on interphase nuclei using a Cy-3 labeled (CCCTAA)3 PNA (Peptide Nuclei Acid) telomeric probe specific for (TTAGGG)n sequences as described in Sgura *et al*.^67^.

Telomere size was estimated by using a dedicated computer program TFL-TELO program (gift from Dr Peter Lansdorp, British Columbia Cancer Centre, Vancouver, Canada). In short, telomeres were identified through segmentation of the DAPI image and the Cy3 image, respectively. Both images were combined and corrected for pixel shifts. The integrated fluorescence intensity for each telomere was calculated after correction for background, based on the values of the surrounding pixels and is proportional to the number of probe molecules that hybridize to the region.

To correct for daily variations in lamp intensity and alignment, images of fluorescent beads (orange beads, size 0.2 mm; Molecular Probes) were acquired and similarly analysed using the IMAGE ANALYSIS computer program, as detailed in Poon and Lansdorp^68^. Telomere fluorescence values were extrapolated from the telomere fluorescence of LY-R and LY-S lymphoma cell lines^69^ of known lengths of 80 and 10 Kb^70^. There was a linear correlation (r^2^ = 0.998) between the fluorescence intensity of the R and S telomeres with a slope of 35.43. The kb length of each telomere was calculated according to the following formula: (Bea1/Bea2) × (TFI/slope) where Bea1 is the fluorescence intensity of beads when LY-R and LY-S were analyzed, Bea2, the fluorescence intensity of beads when sample x was analyzed, and TFI, the unmodified telomere fluorescence intensity in sample x.

Telomere fluorescence intensities were measured at 100× magnification in 50 nuclei per experimental point.

### Measurement of DNA damage: COMET assay

To evaluate DNA damage induction in purified leukocytes, alkaline (pH >13) COMET assay was performed following the Tice-Vasquez procedure^71^. Briefly, at the end of the protocol, leukocytes from acute and chronic (72 h) treatments with H_2_O_2_, as well as proper untreated cultures, were centrifuged for 10 min at 1100 rpm and the pellet re-suspended in PBS. A freshly prepared suspension of cells in 0.75% low melting point agarose (LMA Sigma Chemicals) dissolved in PBS w/o Ca^++^ and Mg^++^ was cast onto microscope slides pre-coated with 0.5% normal melting point agarose (NMA Sigma Chemicals). The cells were then lysed for 1 h at 4 °C in a lysis buffer consisting of 2.5 M NaCl, 100 mM EDTA, 1% Triton X-100, 10% DMSO, 10 mM Tris, pH 10. After the lysis, DNA was allowed to unwind for 40 min in electrophoretic solution consisting of 300 mM NaOH, 1 mM EDTA, pH >13. Electrophoresis was conducted at 4 °C for 30 min at electric field strength 0.73 V/cm (30 mA). The slides were then neutralized with 0.4 M Tris, pH 7.5, fixed with methanol and stored. The slides were examined at 20× magnifications in an Eclipse fluorescence microscope (Nikon, Tokyo, Japan). The images were analysed by Komet 5.5 Image Analysis System (Kinetic Imaging Ltd, Liverpool, UK). Fifty images were randomly selected from each sample and the comet tail DNA was measured. Each experiment was repeated two times. Percentage of DNA in the tail (%DNA tail) was analyzed. The mean value of the % tail DNA in a particular sample was taken as an index of DNA damage in this sample.

### Apoptosis analysis: TUNEL assay

Leukocytes from acute and chronic (72 h) treatments with H_2_O_2_, as well as proper untreated cultures, were rinsed twice with PBS and fixed with paraformaldehyde (4% in PBS) at 4 °C for 1 h. After incubation, cells were permeabilized in 0.1% Triton X100/0.1% sodium citrate solution for 2 min on ice. Cell apoptosis was evaluated by TUNEL assay using the *In situ* Cell Death Detection Kit (Roche Applied Sciences, Germany), according to the manufacturer’s instructions. Apoptosis percentage was evaluated by scoring the number of TUNEL positive nuclei on at least 1000 cells analyzed ×100.

### RNA extraction and Gene Expression Profiling

Gene expression was examined using customized TaqMan Array Plates (Applied Biosystems) (*see supplementary Material*). Expression of 46 different genes involved in Base Excision Repair (n = 14), Damage DNA Binding (n = 14), Double Strand Breaks Repair (n = 5), Cell Cycle and Apoptosis (n = 7), Antioxidants and Defense Systems (n = 6) were targeted for detection by RT-qPCR (Table 2).

Briefly, RNA was isolated by Trizol (Invitrogen) according to the manufacturer’s recommendations. For cDNA synthesis, 10 μL of total RNA (2 μg) was directly processed with the High-Capacity cDNA Archive Kit (#4374966, Applied Biosystems). For each sample, 10 μL of total RNA was added to 10× RT Buffer 2 μL, 25 × dNTP mix 0.8 μL, 10× RT Random primers 2 μL, Reverse Transcriptase 1 μL, RNase Inhibitor 1 μL, Nuclease-free water 3.2 μL to reach a final volume of 20 μL. The reactions were incubated in a GeneAmp PCR System 9700 (Applied Biosystems) at 25 °C for 10 min, 37 °C for 2 h, 85 °C for 5 sec and then at 4 °C. Gene expression data was obtained using TaqMan Low Density Array (TLDA) (Applied Biosystems). One hundred of ng (1 μL) cDNA was used in each sample. 49 μL nuclease-free water and 50 μL 2 × TaqMan Universal PCR Master Mix (#4324018, Applied Biosystems) was added for the Real-Time PCR measurements. This mixture was divided over sample-loading ports of the TLDA. The arrays were centrifuged twice (2 min, 1200 rpm at room temperature). Subsequently, the card was sealed. RT–PCR amplification was performed using an Applied Biosystems Prism 7900HT Sequence Detection System with the following thermal cycler conditions: 2 min at 50 °C and 10 min at 94.5 °C, followed by 40 cycles of 30 sec at 97 °C and 1 min at 59.7 °C. The instrument was connected to Sequence Detector Software (SDS version 2.0; Applied Biosystems) for collection and analysis of data.

### Statistical analysis

All statistical analysis was performed using GraphPad Prism 5.0a (GraphPad Software, Inc). Data are shown as means ± standard deviation (SD). Normality of data distribution was determined using Shapiro–Wilk test, changes in all dependent variables were evaluated using a Two-Way ANOVA. In the case of non-homogeneity of variances revealed by the Mauchly sphericity test (p < 0.05), the Greenhouse–Geisser correction was used to assess significant main effects. Where significant main effects were observed, Bonferroni *post-hoc* correction (p < 0.05) was used to aid interpretation of any interaction. In the “*ex vivo*” H_2_O_2_-treated protocols, the telomere length was calculated for each subjects as fold change by the formula: H_2_O_2_-treated LTL/untreated LTL.

An unpaired t-test was used to analyse the differences in gene expression data, obtained by RT-qPCR array, between UT- and TR-T2D patients; *p < 0.05 and **p < 0.01 were considered to indicate significant and highly significant values, respectively.

### Data Availability

The datasets generated and analysed during the current study are available from the corresponding author on reasonable request.

## Electronic supplementary material

Supplementary Table S1
