# Supplementary material for: Regular exercise participation improves genomic stability in diabetic patients: an exploratory study to analyse telomere length and DNA damage
Source: Sci Rep. 2017 Jun 23;7:4137. doi: 10.1038/s41598-017-04448-4 (PMC5482873; doi:10.1038/s41598-017-04448-4)
Supplement: Supplementary file 1 — Supplementary Table S1 [file 41598_2017_4448_MOESM1_ESM.doc]

**Regular exercise participation improves genomic stability in diabetic patients: an exploratory study to analyse telomere length and DNA damage**

Ivan Dimauro, Antonella Sgura, Monica Pittaluga, Fiorenza Magi, Cristina Fantini, Rosa Mancinelli, Antonio Sgadari, Stefania Fulle, Daniela Caporossi

**Supplementary Table S1. List of genes analyzed by quantitative RT-PCR array.**

| **Gene ID** | **Assay ID** | **Gene Symbol** | **Name** |
| --- | --- | --- | --- |
| 2597 | Hs99999905_m1 | GAPDH | glyceraldehyde-3-phosphate dehydrogenase |
| 328 | Hs00172396_m1 | APEX1 | APEX nuclease (multifunctional DNA repair enzyme) 1 |
| [2237](http://www.ncbi.nlm.nih.gov/sites/entrez?cmd=retrieve&db=gene&list_uids=4088&dopt=full_report) | Hs00748727_s1 | FEN1 | flap structure-specific endonuclease 1 |
| [3978](http://www.ncbi.nlm.nih.gov/sites/entrez?cmd=retrieve&db=gene&list_uids=51588&dopt=full_report) | Hs01553527_m1 | LIG1 | DNA ligase 1 |
| [4350](http://www.ncbi.nlm.nih.gov/sites/entrez?cmd=retrieve&db=gene&list_uids=5518&dopt=full_report) | Hs01012594_m1 | MPG | N-methylpurine DNA glycosylase alpha isoform |
| 3980 | Hs00242692_m1 | LIG3 | ligase III, DNA, ATP-dependent |
| 5111 | Hs00952870_g1 | PCNA | proliferating cell nuclear antigen |
| 4968 | Hs00213454_m1 | OGG1 | 8-oxoguanine DNA glycosylase |
| [142](http://www.ncbi.nlm.nih.gov/sites/entrez?cmd=retrieve&db=gene&list_uids=25793&dopt=full_report) | Hs00914307_g1 | PARP1 | poly(ADP-ribose) polymerase 1 |
| [11284](http://www.ncbi.nlm.nih.gov/sites/entrez?cmd=retrieve&db=gene&list_uids=10015&dopt=full_report) | Hs00200647_m1 | PNKP | polynucleotide kinase 3'-phosphatase |
| [5423](http://www.ncbi.nlm.nih.gov/sites/entrez?cmd=retrieve&db=gene&list_uids=7015&dopt=full_report) | Hs00160263_m1 | POLB | polymerase (DNA directed), beta |
| [6996](http://www.ncbi.nlm.nih.gov/sites/entrez?cmd=retrieve&db=gene&list_uids=60&dopt=full_report) | Hs00702322_s1 | TDG | thymine DNA glycosylase |
| 7374 | Hs00422172_m1 | UNG | uracil DNA glycosylase |
| 10309 | Hs00221731_m1 | CCNO | cyclin O |
| 7515 | Hs00173077_m1 | XRCC1 | X-ray repair cross-complementing protein 1 |
| 472 | Hs01112317_g1 | ATM | Ataxia telangiectasia mutated |
| 1111 | Hs00176236_m1 | CHEK1 | checkpoint kinase 1 |
| 1647 | Hs00169255_m1 | GADD45A | growth arrest and DNA damage inducible alpha |
| 596 | Hs00236808_s1 | BCL2 | BCL2, apoptosis regulator |
| 581 | Hs00180269_m1 | BAX | BCL2 associated X, apoptosis regulator |
| [7157](http://www.ncbi.nlm.nih.gov/sites/entrez?cmd=retrieve&db=gene&list_uids=7157&dopt=full_report) | Hs99999147_m1 | TP53 | tumor protein p53 |
| 2876 | Hs00829989_gH | GPX1 | glutathione peroxidase 1 |
| 847 | Hs00156308_m1 | CAT | catalase |
| 2936 | Hs00167317_m1 | GSR | glutathione-disulfide reductase |
| 6647 | Hs00916176_m1 | SOD1 | superoxide dismutase 1, soluble |
| 55074 | Hs00250562_m1 | OXR1 | oxidation resistance 1 |
| 3308 | Hs00382884_m1 | HSPA4 | heat shock protein family A (Hsp70) member 4 |
| 672 | Hs00173233_m1 | BRCA1 | BRCA1, DNA repair associated |
| 2067 | Hs01012158_m1 | ERCC1 | ERCC excision repair 1, endonuclease non-catalytic subunit |
| 2068 | Hs00361161_m1 | ERCC2 | ERCC excision repair 2, TFIIH core complex helicase subunit |
| 3014 | Hs00266783_s1 | H2AFX | H2A histone family member X |
| 4436 | Hs00953523_m1 | MSH2 | mutS homolog 2 |
| 4437 | Hs00989003_m1 | MSH3 | mutS homolog 3 |
| 5810 | Hs00421891_g1 | RAD1 | RAD1 checkpoint DNA exonuclease |
| 5888 | Hs00153418_m1 | RAD51 | RAD51 recombinase |
| 5889 | Hs00427442_m1 | RAD51C | RAD51 paralog C |
| 7516 | Hs00538799_m1 | XRCC2 | X-ray repair cross complementing 2 |
| 7507 | Hs00166045_m1 | XPA | XPA, DNA damage recognition and repair factor |
| 7508 | Hs00190295_m1 | XPC | XPC complex subunit, DNA damage recognition and repair factor |
| 4913 | Hs00267385_m1 | NTHL1 | nth like DNA glycosylase 1 |
| 4595 | Hs01014856_m1 | MUTYH | mutY DNA glycosylase |
| 675 | Hs00609060_m1 | BRCA2 | BRCA2, DNA repair associated |
| 4361 | Hs00967449_g1 | MRE11A | MRE11 homolog, double strand break repair nuclease |
| 5893 | Hs00172536_m1 | RAD52 | RAD52 homolog, DNA repair protein |
| 5591 | Hs00179161_m1 | PRKDC | protein kinase, DNA-activated, catalytic polypeptide |
| 2547 | Hs01922652_g1 | XRCC6 | X-ray repair cross complementing 6 |
| 100008588 | Hs99999901_s1 | 18S | RNA, 18S ribosomal 5 |
| 836 | Hs00234387_m1 | CASP3 | caspase 3 |
